# Bis{*S*-benzyl 3-[(6-methyl­pyridin-2-yl)methyl­idene]dithio­carbazato}nickel(II)

**DOI:** 10.1107/S1600536812017333

**Published:** 2012-04-25

**Authors:** Thahira Begum S. A. Ravoof, Siti Aminah Omar, Mohamed Ibrahim Mohamed Tahir, Karen A. Crouse

**Affiliations:** a Department of Chemistry, Faculty of Science, Universiti Putra Malaysia, 43400 UPM, Serdang, Selangor, Malaysia

## Abstract

The asymmetric unit of the title compound, [Ni(C_15_H_14_N_3_S_2_)_2_], consists of two independent mol­ecules with similar configurations. Each Ni^2+^ cation is coordinated in a *cis*-mode by two tridentate *N*,*N*′,*S*-chelating Schiff base ligands, creating a distorted octa­hedron [the smallest angle being 77.57 (7)° and the widest being 168.97 (7)° for one mol­ecule, and 78.04 (7) and 167.55 (7)° for the second mol­ecule]. The dihedral angle between the mean coordination planes of the two ligands is 86.76 (7)° for one and 89.99 (7)° for the second mol­ecule. π–π inter­actions between neighbouring pyridine rings with plane-to-plane distances of 3.540 (1) and 3.704 (1) Å are observed.

## Related literature

For background to the coordination chemistry of hydrazine carbodithio­ates, see: Ravoof *et al.* (2010 ▶). For the synthesis, see: Ravoof *et al.* (2004 ▶). For related structures, see: Ali *et al.* (1997 ▶, 1999 ▶); Omar *et al.* (2012 ▶).
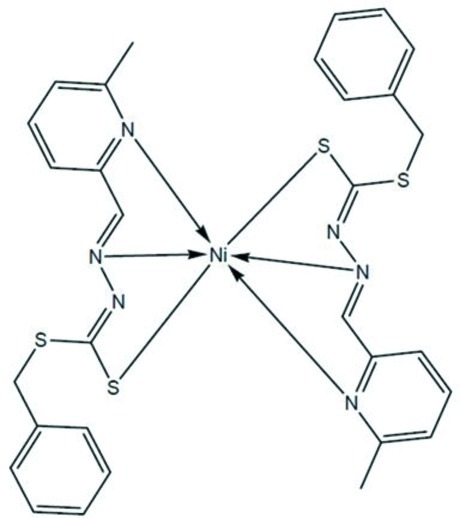


## Experimental

### 

#### Crystal data

[Ni(C_15_H_14_N_3_S_2_)_2_]
*M* *_r_* = 659.57
Triclinic, 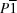

*a* = 12.3544 (9) Å
*b* = 15.6411 (6) Å
*c* = 16.9333 (10) Å
α = 69.520 (5)°
β = 87.516 (5)°
γ = 89.446 (5)°
*V* = 3062.4 (3) Å^3^
*Z* = 4
Mo *K*α radiation
μ = 0.94 mm^−1^
*T* = 150 K
0.16 × 0.13 × 0.11 mm

#### Data collection

Oxford Diffraction Gemini diffractometer
Absorption correction: multi-scan (*CrysAlis PRO*; Agilent, 2011 ▶) *T*_min_ = 0.88, *T*_max_ = 0.90
23348 measured reflections
13743 independent reflections
11306 reflections with *I* > 2σ(*I*)
*R*_int_ = 0.035

#### Refinement

*R*[*F* ^2^ > 2σ(*F* ^2^)] = 0.038
*wR*(*F* ^2^) = 0.087
*S* = 0.97
13696 reflections
739 parameters
H-atom parameters constrained
Δρ_max_ = 0.52 e Å^−3^
Δρ_min_ = −0.51 e Å^−3^

### 

Data collection: *CrysAlis PRO* (Agilent, 2011 ▶); cell refinement: *CrysAlis PRO*; data reduction: *CrysAlis PRO*; program(s) used to solve structure: *SIR92* (Altomare *et al.*, 1994 ▶); program(s) used to refine structure: *CRYSTALS* (Betteridge *et al.*, 2003 ▶); molecular graphics: *CAMERON* (Watkin *et al.*, 1996 ▶); software used to prepare material for publication: *CRYSTALS*.

## Supplementary Material

Crystal structure: contains datablock(s) global, I. DOI: 10.1107/S1600536812017333/wm2622sup1.cif

Structure factors: contains datablock(s) I. DOI: 10.1107/S1600536812017333/wm2622Isup2.hkl

Additional supplementary materials:  crystallographic information; 3D view; checkCIF report

## Figures and Tables

**Table 1:** Selected bond lengths (Å)

Ni1—N202	2.0139 (18)
Ni1—N102	2.0173 (17)
Ni1—N115	2.1761 (18)
Ni1—N215	2.1881 (18)
Ni1—S105	2.4062 (6)
Ni1—S205	2.4158 (6)
Ni2—N302	2.0085 (18)
Ni2—N402	2.0156 (18)
Ni2—N315	2.1604 (17)
Ni2—N415	2.1770 (18)
Ni2—S405	2.4202 (6)
Ni2—S305	2.4263 (6)

**Fig. 1.**
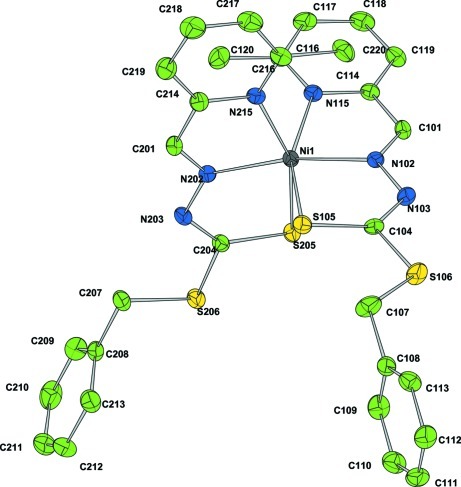
The title compound with displacement ellipsoids drawn at the 50% probability level. Only one of the molecules in the asymmetric unit is shown.

**Fig. 2.**
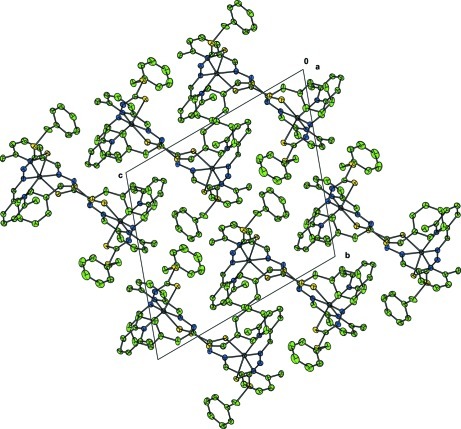
Molecular packing diagram of the title compound viewed along the a axis. Hydrogen atoms are omitted for clarity.

## supplementary crystallographic information

### Comment

The title compound, [Ni(C_15_H_14_N_3_S_2_)_2_], was preferentially formed by elimination of the saccharinate anion from nickel(II) saccharinate during an attempt to introduce *S*-benzyl-beta-*N*-(6-methylpyrid-2-yl) methylenedithiocarbazate (for details see experimental part). More background on the coordination chemistry of hydrazine carbodithioates is given by Ravoof *et al.* (2010).

In the structure of the title compound two independent molecules are in the asymmetric unit with similar configurations. The Ni^2+^ cations are coordinated by two tridentate ligands in form of distorted NiN_4_S_2_ octahedra with angles varying from 77.57 (7)° to 168.97 (7)°. The ligands are coordinating to the Ni^2+^ ions in their deprotonated mercaptide forms *via* the pyridine nitrogen atoms, the azomethine nitrogen atoms and the thiolate sulfur atoms. The deprotonation of the ligands is accompanied by their tautomerism to the iminothiolate forms. While coordinating in the iminothiolate form, the negative charge generated upon deprotonation is delocalized in the C—N—N—C system as observed by their intermediate bond lengths C(1-4)04—N(1-4)03 = 1.310 (3) to 1.314 (3) Å, N(1-4)03—N(1-4)02 = 1.382 (3) to 1.391 (3) Å and N(1-4)02—C(1-4)01 = 1.285 (3) to 1.289 (3) Å. Similar bond angles and bond lengths have been observed in the monohydrate Ni(II) complex of the same 6-methylpyridine-2-aldehyde Schiff base with C—N bonds ranging from 1.281 (3) Å to 1.358 (3) Å and N—N bonds ranging from 1.379 (2) to 1.386 (2) Å (Omar *et al.*, 2012)

The two ligands coordinate to the nickel(II) ion in a *cis* mode (Fig. 1). Similar configurations have been observed in other bis-ligand metal complexes of related *NNS*-tridentate ligands (Ali *et al.*, 1997, 1999). The angle between the planes defined by S205—C204—N203—N202—C201—C214—N215 (minimum deviation from the mean plane: 0.003 Å and maximum deviation 0.04 Å), and S105—C104—N103—N102—C101—C114—N115 (0.001 and 0.094 Å) is 86.76 (7)°. The angle between the corresponding planes in the molecule containing Ni2 is perfectly orthogonal with a value of 89.99 (7)°.

The bond lengths Ni1—S [2.4062 (6) – 2.4263 (6) Å], Ni1—N_py_ [2.1604 (17)—2.1881 (18) Å] and Ni1—N_imine_ [2.0085 (18)—2.0173 (17) Å] compare well with related complexes with octahedrally coordinated Ni^2+^ cations containing 6-methylpyridine-2-aldehyde Schiff bases of *S*—*R*-dithiocarbazate (*R* = methyl or benzyl), where Ni—S bond lengths of 2.420 (6)–2.426 (5) Å, Ni-N_py_ bond lengths of 2.166 (2)–2.179 (2) Å and Ni-N_imine_ bond lengths of 2.016 (2)–2.019 (2) Å are observed (Omar *et al.*, 2012; Ali *et al.*, 1997, 1999).

None of the bond angles in the complex conform to the ideal values expected of a regular octahedral geometry, a trend that was observed in other related complexes indicating that distortion from ideal octahedral geometry is a common phenomenon in six-coordinate metal complexes of Schiff base ligands derived from dithiocarbazic acid and thiosemicarbazone ligands (Ali *et al.*, 1997).

The packing of the structure (Fig. 2) is dominated by π—π interactions between neighbouring pyridine rings with plane-to-plane distances of 3.540 (1) and 3.704 (1) Å.

### Experimental

Nickel(II) saccharinate, [Ni(sac)_2_(H_2_O)_4_]^.^2H_2_O was prepared using a similar procedure used for the synthesis of Cu(II) saccharinate] (Ravoof *et al.*, 2004). The 6-methyl-2-pyridine carboxaldehyde Schiff base of *S*-benzyldithiocarbazate was synthesized following the procedure given by Ali *et al.* (1997). [Ni(sac)_2_(H_2_O)_4_]^.^2H_2_O (0.001 mol) was dissolved in ethanol (25 ml) and was mixed with a solution of the appropriate Schiff base (0.001 mol) in ethanol (50 ml). The resulting mixture was heated on a water bath until the volume reduced to 30 ml. On standing overnight, the mixture yielded crystals which were filtered off, washed with ethanol and dried in a desiccator over anhydrous silica gel. Upon recrystallization from acetonitrile, the solution yielded blackish green crystals suitable for X-ray analysis.

### Refinement

The H atoms were all located in a difference map, but those attached to C atoms were repositioned geometrically. The H atoms were initially refined with soft restraints on the bond lengths and angles to regularize their geometry (C—H in the range 0.93–0.98, N—H in the range 0.86–0.89 Å) and *U*_iso_(H)(in the range 1.2–1.5 times *U*_eq_ of the parent atom), after which the positions were refined with riding constraints.

### Crystal data

**Table:**

[Ni(C_15_H_14_N_3_S_2_)_2_]	*Z* = 4
*M**_r_* = 659.57	*F*(000) = 1368
Triclinic, *P*1	*D*_x_ = 1.430 Mg m^−^^3^
Hall symbol: -P 1	Mo *K*α radiation, λ = 0.71073 Å
*a* = 12.3544 (9) Å	Cell parameters from 10517 reflections
*b* = 15.6411 (6) Å	θ = 2–29°
*c* = 16.9333 (10) Å	µ = 0.94 mm^−^^1^
α = 69.520 (5)°	*T* = 150 K
β = 87.516 (5)°	Plate, green–black
γ = 89.446 (5)°	0.16 × 0.13 × 0.11 mm
*V* = 3062.4 (3) Å^3^	

### Data collection

**Table:**

Oxford Diffraction Gemini diffractometer	11306 reflections with *I* > 2σ(*I*)
Graphite monochromator	*R*_int_ = 0.035
ω scans	θ_max_ = 28.8°, θ_min_ = 2.2°
Absorption correction: multi-scan (*CrysAlis PRO*; Agilent, 2011)	*h* = −14→16
*T*_min_ = 0.88, *T*_max_ = 0.90	*k* = −20→20
23348 measured reflections	*l* = −19→22
13743 independent reflections	

### Refinement

**Table:**

Refinement on *F*^2^	Primary atom site location: structure-invariant direct methods
Least-squares matrix: full	Hydrogen site location: difference Fourier map
*R*[*F*^2^ > 2σ(*F*^2^)] = 0.038	H-atom parameters constrained
*wR*(*F*^2^) = 0.087	Method = Modified Sheldrick *w* = 1/[σ^2^(*F*^2^) + (0.03*P*)^2^ + 1.88*P*], where *P* = (max(*F*_o_^2^,0) + 2*F*_c_^2^)/3
*S* = 0.97	(Δ/σ)_max_ = 0.001
13696 reflections	Δρ_max_ = 0.52 e Å^−^^3^
739 parameters	Δρ_min_ = −0.51 e Å^−^^3^
0 restraints	

### Special details

**Table:**

Experimental. The crystal was placed in the cold stream of an Oxford Cryosystems open-flow nitrogen cryostat (Cosier & Glazer, 1986) with a nominal stability of 0.1 K.Cosier, J. & Glazer, A. M., 1986. J. Appl. Cryst. 105–107.

### Fractional atomic coordinates and isotropic or equivalent isotropic displacement parameters (Å^2^)

**Table:**

	*x*	*y*	*z*	*U*_iso_*/*U*_eq_	
Ni1	1.12017 (2)	0.224568 (18)	0.553643 (17)	0.0162	
Ni2	0.61915 (2)	−0.219821 (18)	0.928595 (17)	0.0174	
C101	1.24970 (17)	0.22929 (14)	0.40808 (14)	0.0187	
N102	1.24657 (14)	0.26047 (12)	0.46896 (11)	0.0170	
N103	1.33713 (14)	0.30674 (12)	0.47823 (12)	0.0198	
C104	1.32700 (17)	0.33182 (14)	0.54446 (14)	0.0181	
S105	1.21898 (4)	0.31606 (4)	0.61479 (4)	0.0203	
S106	1.44856 (5)	0.38628 (4)	0.55348 (4)	0.0282	
C107	1.4177 (2)	0.43003 (19)	0.63824 (17)	0.0330	
C108	1.51814 (18)	0.42759 (16)	0.68628 (15)	0.0234	
C109	1.5573 (2)	0.34505 (16)	0.73939 (16)	0.0294	
C110	1.6484 (2)	0.34216 (18)	0.78491 (16)	0.0334	
C111	1.7012 (2)	0.42240 (19)	0.77766 (16)	0.0328	
C112	1.6632 (2)	0.50489 (17)	0.72499 (16)	0.0305	
C113	1.57170 (19)	0.50737 (16)	0.67944 (15)	0.0264	
C114	1.15435 (17)	0.17925 (14)	0.39935 (14)	0.0184	
N115	1.07218 (14)	0.16983 (12)	0.45855 (11)	0.0179	
C116	0.98108 (18)	0.12688 (14)	0.45174 (14)	0.0212	
C117	0.97130 (19)	0.09107 (15)	0.38745 (15)	0.0261	
C118	1.0556 (2)	0.09912 (16)	0.32930 (15)	0.0281	
C119	1.14910 (19)	0.14487 (15)	0.33469 (14)	0.0237	
C120	0.88963 (19)	0.11928 (18)	0.51433 (16)	0.0309	
C201	0.93232 (18)	0.22883 (15)	0.65720 (14)	0.0226	
N202	1.01608 (14)	0.18004 (12)	0.65462 (11)	0.0192	
N203	1.03247 (15)	0.10453 (12)	0.72600 (11)	0.0213	
C204	1.12170 (17)	0.06187 (14)	0.71794 (13)	0.0184	
S205	1.21361 (4)	0.08743 (4)	0.63423 (3)	0.0195	
S206	1.15372 (5)	−0.03460 (4)	0.80431 (4)	0.0238	
C207	1.0465 (2)	−0.03433 (18)	0.88156 (15)	0.0311	
C208	1.07025 (18)	−0.10703 (16)	0.96397 (15)	0.0245	
C209	1.0385 (2)	−0.19663 (18)	0.98112 (17)	0.0332	
C210	1.0589 (2)	−0.26306 (18)	1.05803 (19)	0.0395	
C211	1.1103 (2)	−0.23981 (19)	1.11871 (17)	0.0396	
C212	1.1437 (2)	−0.1512 (2)	1.10169 (16)	0.0384	
C213	1.1249 (2)	−0.08537 (18)	1.02436 (16)	0.0302	
C214	0.91452 (18)	0.30912 (15)	0.58346 (14)	0.0223	
N215	0.99131 (14)	0.32631 (12)	0.51951 (11)	0.0191	
C216	0.97919 (19)	0.40004 (15)	0.45035 (14)	0.0233	
C217	0.8910 (2)	0.45799 (16)	0.44331 (16)	0.0306	
C218	0.8139 (2)	0.44024 (17)	0.50790 (17)	0.0337	
C219	0.8249 (2)	0.36452 (17)	0.57980 (16)	0.0300	
C220	1.0647 (2)	0.41814 (17)	0.38081 (15)	0.0306	
C301	0.41885 (18)	−0.17359 (16)	0.84699 (15)	0.0236	
N302	0.51384 (14)	−0.14061 (12)	0.84787 (11)	0.0190	
N303	0.53850 (15)	−0.05580 (12)	0.78730 (12)	0.0231	
C304	0.63767 (18)	−0.02988 (14)	0.79341 (14)	0.0202	
S305	0.73372 (5)	−0.08661 (4)	0.86234 (4)	0.0240	
S306	0.68046 (5)	0.07864 (4)	0.72420 (4)	0.0289	
C307	0.5694 (2)	0.11920 (16)	0.65294 (15)	0.0283	
C308	0.58840 (18)	0.10579 (15)	0.56976 (15)	0.0232	
C309	0.62070 (17)	0.02120 (15)	0.56667 (15)	0.0232	
C310	0.63576 (18)	0.00845 (17)	0.49040 (16)	0.0271	
C311	0.61756 (19)	0.07902 (18)	0.41605 (16)	0.0310	
C312	0.5841 (2)	0.16278 (18)	0.41823 (16)	0.0350	
C313	0.5701 (2)	0.17619 (16)	0.49494 (16)	0.0295	
C314	0.39307 (18)	−0.26258 (16)	0.91029 (14)	0.0228	
N315	0.47294 (14)	−0.30143 (12)	0.96410 (11)	0.0196	
C316	0.45432 (18)	−0.38407 (15)	1.02323 (14)	0.0229	
C317	0.3556 (2)	−0.42943 (17)	1.02960 (16)	0.0302	
C318	0.2753 (2)	−0.38965 (19)	0.97532 (17)	0.0357	
C319	0.2935 (2)	−0.30439 (18)	0.91437 (16)	0.0329	
C320	0.5417 (2)	−0.42539 (16)	1.08248 (16)	0.0298	
C401	0.77504 (18)	−0.27479 (15)	1.05556 (14)	0.0220	
N402	0.74818 (14)	−0.28841 (12)	0.98833 (11)	0.0188	
N403	0.82005 (14)	−0.33770 (12)	0.95551 (12)	0.0209	
C404	0.78566 (17)	−0.34309 (14)	0.88505 (14)	0.0192	
S405	0.66807 (4)	−0.30251 (4)	0.83542 (4)	0.0217	
S406	0.86870 (5)	−0.39769 (4)	0.83081 (4)	0.0260	
C407	0.99658 (19)	−0.41463 (19)	0.88374 (15)	0.0304	
C408	1.08930 (18)	−0.40465 (16)	0.82007 (15)	0.0231	
C409	1.1586 (2)	−0.47663 (18)	0.82676 (17)	0.0336	
C410	1.2456 (2)	−0.4667 (2)	0.76938 (19)	0.0450	
C411	1.2633 (2)	−0.3847 (2)	0.7050 (2)	0.0476	
C412	1.1951 (2)	−0.3128 (2)	0.69777 (19)	0.0430	
C413	1.1083 (2)	−0.32230 (17)	0.75497 (17)	0.0316	
C414	0.70273 (18)	−0.22005 (15)	1.08827 (14)	0.0213	
N415	0.61173 (14)	−0.18980 (12)	1.04492 (11)	0.0189	
C416	0.54162 (18)	−0.13981 (15)	1.07363 (14)	0.0209	
C417	0.5635 (2)	−0.11748 (15)	1.14437 (15)	0.0258	
C418	0.6563 (2)	−0.14756 (16)	1.18746 (15)	0.0275	
C419	0.72743 (19)	−0.20058 (16)	1.15916 (15)	0.0255	
C420	0.43978 (19)	−0.10927 (17)	1.02746 (16)	0.0274	
H1011	1.3100	0.2376	0.3705	0.0218*	
H1072	1.3922	0.4940	0.6133	0.0421*	
H1071	1.3595	0.3917	0.6738	0.0413*	
H1091	1.5210	0.2897	0.7451	0.0348*	
H1101	1.6753	0.2854	0.8213	0.0398*	
H1111	1.7645	0.4206	0.8093	0.0389*	
H1121	1.6989	0.5595	0.7209	0.0368*	
H1131	1.5449	0.5653	0.6425	0.0325*	
H1171	0.9045	0.0616	0.3848	0.0312*	
H1181	1.0495	0.0725	0.2855	0.0342*	
H1191	1.2086	0.1532	0.2948	0.0291*	
H1202	0.8349	0.0802	0.5073	0.0462*	
H1201	0.9132	0.0934	0.5716	0.0460*	
H1203	0.8594	0.1795	0.5047	0.0461*	
H2011	0.8839	0.2125	0.7052	0.0276*	
H2072	1.0478	0.0258	0.8875	0.0392*	
H2071	0.9778	−0.0466	0.8606	0.0391*	
H2091	0.9999	−0.2119	0.9404	0.0417*	
H2101	1.0368	−0.3238	1.0683	0.0477*	
H2111	1.1219	−0.2851	1.1719	0.0486*	
H2121	1.1784	−0.1346	1.1436	0.0463*	
H2131	1.1482	−0.0237	1.0124	0.0381*	
H2171	0.8857	0.5096	0.3926	0.0370*	
H2181	0.7541	0.4795	0.5034	0.0418*	
H2191	0.7715	0.3488	0.6262	0.0367*	
H2202	1.0477	0.4720	0.3349	0.0482*	
H2201	1.1340	0.4259	0.4014	0.0487*	
H2203	1.0703	0.3678	0.3604	0.0480*	
H3011	0.3670	−0.1401	0.8067	0.0297*	
H3072	0.5618	0.1843	0.6433	0.0349*	
H3071	0.5017	0.0874	0.6808	0.0346*	
H3091	0.6321	−0.0276	0.6183	0.0286*	
H3101	0.6575	−0.0505	0.4899	0.0321*	
H3111	0.6267	0.0696	0.3641	0.0389*	
H3121	0.5708	0.2124	0.3673	0.0426*	
H3131	0.5467	0.2344	0.4973	0.0374*	
H3171	0.3450	−0.4882	1.0721	0.0364*	
H3181	0.2083	−0.4208	0.9809	0.0415*	
H3191	0.2386	−0.2745	0.8759	0.0412*	
H3202	0.5196	−0.4852	1.1187	0.0452*	
H3201	0.5568	−0.3885	1.1154	0.0457*	
H3203	0.6068	−0.4298	1.0512	0.0448*	
H4011	0.8408	−0.2998	1.0839	0.0276*	
H4072	0.9980	−0.4751	0.9284	0.0388*	
H4071	1.0030	−0.3676	0.9090	0.0385*	
H4091	1.1463	−0.5344	0.8717	0.0420*	
H4101	1.2922	−0.5161	0.7749	0.0549*	
H4111	1.3212	−0.3788	0.6656	0.0560*	
H4121	1.2070	−0.2565	0.6536	0.0518*	
H4131	1.0630	−0.2716	0.7493	0.0391*	
H4171	0.5126	−0.0822	1.1628	0.0322*	
H4181	0.6708	−0.1319	1.2347	0.0350*	
H4191	0.7925	−0.2246	1.1880	0.0334*	
H4202	0.3978	−0.0733	1.0537	0.0416*	
H4201	0.3984	−0.1625	1.0303	0.0404*	
H4203	0.4563	−0.0724	0.9693	0.0401*	

### Atomic displacement parameters (Å^2^)

**Table:**

	*U*^11^	*U*^22^	*U*^33^	*U*^12^	*U*^13^	*U*^23^
Ni1	0.01624 (14)	0.01809 (14)	0.01484 (14)	−0.00187 (10)	0.00013 (11)	−0.00648 (11)
Ni2	0.01612 (14)	0.02080 (14)	0.01658 (14)	−0.00033 (11)	−0.00106 (11)	−0.00804 (12)
C101	0.0200 (11)	0.0201 (10)	0.0169 (11)	−0.0011 (8)	0.0025 (9)	−0.0079 (9)
N102	0.0164 (9)	0.0185 (9)	0.0160 (9)	−0.0023 (7)	−0.0016 (7)	−0.0057 (8)
N103	0.0176 (9)	0.0211 (9)	0.0225 (10)	−0.0059 (7)	−0.0010 (8)	−0.0097 (8)
C104	0.0181 (11)	0.0160 (10)	0.0207 (11)	−0.0001 (8)	−0.0054 (9)	−0.0065 (9)
S105	0.0217 (3)	0.0228 (3)	0.0195 (3)	−0.0022 (2)	−0.0007 (2)	−0.0110 (2)
S106	0.0221 (3)	0.0372 (3)	0.0338 (3)	−0.0084 (2)	−0.0006 (3)	−0.0229 (3)
C107	0.0255 (13)	0.0455 (15)	0.0424 (16)	0.0034 (11)	−0.0090 (11)	−0.0326 (14)
C108	0.0220 (11)	0.0308 (12)	0.0213 (12)	−0.0045 (9)	0.0000 (9)	−0.0141 (10)
C109	0.0322 (13)	0.0245 (12)	0.0330 (14)	−0.0068 (10)	0.0025 (11)	−0.0123 (11)
C110	0.0363 (15)	0.0329 (14)	0.0267 (13)	0.0060 (11)	−0.0033 (11)	−0.0051 (12)
C111	0.0262 (13)	0.0497 (16)	0.0287 (13)	0.0013 (12)	−0.0072 (11)	−0.0209 (13)
C112	0.0284 (13)	0.0356 (14)	0.0338 (14)	−0.0105 (11)	−0.0001 (11)	−0.0198 (12)
C113	0.0330 (13)	0.0254 (12)	0.0223 (12)	−0.0035 (10)	−0.0032 (10)	−0.0098 (10)
C114	0.0212 (11)	0.0168 (10)	0.0177 (11)	0.0000 (8)	−0.0030 (9)	−0.0065 (9)
N115	0.0196 (9)	0.0174 (9)	0.0175 (9)	−0.0016 (7)	−0.0025 (7)	−0.0067 (8)
C116	0.0218 (11)	0.0175 (10)	0.0214 (11)	−0.0016 (9)	−0.0050 (9)	−0.0025 (9)
C117	0.0269 (12)	0.0241 (12)	0.0284 (13)	−0.0070 (10)	−0.0076 (10)	−0.0095 (11)
C118	0.0362 (14)	0.0262 (12)	0.0252 (12)	−0.0032 (10)	−0.0081 (11)	−0.0124 (11)
C119	0.0302 (13)	0.0224 (11)	0.0192 (11)	−0.0027 (9)	−0.0006 (10)	−0.0079 (10)
C120	0.0238 (12)	0.0378 (14)	0.0333 (14)	−0.0097 (11)	−0.0001 (11)	−0.0151 (12)
C201	0.0193 (11)	0.0285 (12)	0.0192 (11)	0.0021 (9)	0.0027 (9)	−0.0078 (10)
N202	0.0177 (9)	0.0211 (9)	0.0175 (9)	−0.0011 (7)	−0.0013 (7)	−0.0051 (8)
N203	0.0227 (10)	0.0215 (9)	0.0153 (9)	0.0011 (8)	0.0002 (8)	−0.0009 (8)
C204	0.0194 (11)	0.0202 (10)	0.0158 (10)	−0.0023 (8)	−0.0005 (9)	−0.0065 (9)
S205	0.0197 (3)	0.0206 (3)	0.0185 (3)	0.0005 (2)	0.0016 (2)	−0.0074 (2)
S206	0.0234 (3)	0.0244 (3)	0.0193 (3)	0.0040 (2)	0.0010 (2)	−0.0025 (2)
C207	0.0259 (13)	0.0417 (15)	0.0180 (12)	0.0081 (11)	0.0028 (10)	−0.0014 (11)
C208	0.0191 (11)	0.0298 (12)	0.0200 (11)	0.0032 (9)	0.0032 (9)	−0.0037 (10)
C209	0.0293 (13)	0.0369 (14)	0.0316 (14)	−0.0003 (11)	−0.0007 (11)	−0.0100 (12)
C210	0.0332 (15)	0.0271 (13)	0.0463 (17)	0.0006 (11)	0.0078 (13)	0.0007 (13)
C211	0.0332 (15)	0.0447 (16)	0.0244 (13)	0.0103 (12)	−0.0009 (11)	0.0085 (13)
C212	0.0356 (15)	0.0555 (18)	0.0212 (13)	0.0065 (13)	−0.0058 (11)	−0.0095 (13)
C213	0.0309 (13)	0.0332 (13)	0.0252 (13)	0.0015 (11)	0.0022 (11)	−0.0091 (11)
C214	0.0213 (11)	0.0254 (12)	0.0208 (11)	0.0014 (9)	−0.0005 (9)	−0.0089 (10)
N215	0.0202 (9)	0.0203 (9)	0.0171 (9)	0.0000 (7)	−0.0031 (8)	−0.0065 (8)
C216	0.0292 (12)	0.0213 (11)	0.0199 (11)	0.0001 (9)	−0.0041 (10)	−0.0073 (10)
C217	0.0375 (14)	0.0255 (12)	0.0275 (13)	0.0094 (10)	−0.0077 (11)	−0.0070 (11)
C218	0.0346 (14)	0.0329 (14)	0.0352 (15)	0.0155 (11)	−0.0066 (12)	−0.0137 (12)
C219	0.0270 (13)	0.0351 (14)	0.0283 (13)	0.0081 (10)	0.0007 (11)	−0.0122 (12)
C220	0.0386 (15)	0.0284 (13)	0.0201 (12)	0.0045 (11)	0.0004 (11)	−0.0028 (11)
C301	0.0183 (11)	0.0306 (12)	0.0225 (12)	0.0028 (9)	−0.0032 (9)	−0.0100 (10)
N302	0.0190 (9)	0.0210 (9)	0.0171 (9)	0.0006 (7)	0.0005 (7)	−0.0068 (8)
N303	0.0264 (10)	0.0218 (9)	0.0187 (10)	0.0005 (8)	0.0010 (8)	−0.0042 (8)
C304	0.0252 (12)	0.0200 (11)	0.0169 (11)	−0.0018 (9)	0.0022 (9)	−0.0085 (9)
S305	0.0213 (3)	0.0259 (3)	0.0241 (3)	−0.0049 (2)	−0.0021 (2)	−0.0075 (3)
S306	0.0362 (3)	0.0235 (3)	0.0250 (3)	−0.0068 (2)	0.0009 (3)	−0.0061 (3)
C307	0.0351 (14)	0.0244 (12)	0.0232 (12)	0.0073 (10)	0.0011 (11)	−0.0062 (11)
C308	0.0200 (11)	0.0248 (12)	0.0238 (12)	0.0011 (9)	0.0028 (9)	−0.0078 (10)
C309	0.0199 (11)	0.0237 (11)	0.0248 (12)	0.0001 (9)	0.0021 (9)	−0.0072 (10)
C310	0.0212 (12)	0.0302 (13)	0.0318 (13)	−0.0007 (10)	0.0033 (10)	−0.0137 (11)
C311	0.0266 (13)	0.0451 (15)	0.0247 (13)	−0.0033 (11)	0.0029 (10)	−0.0168 (12)
C312	0.0338 (14)	0.0392 (15)	0.0239 (13)	0.0018 (11)	−0.0009 (11)	−0.0008 (12)
C313	0.0319 (13)	0.0240 (12)	0.0276 (13)	0.0029 (10)	0.0035 (11)	−0.0033 (11)
C314	0.0195 (11)	0.0299 (12)	0.0205 (11)	−0.0029 (9)	−0.0002 (9)	−0.0107 (10)
N315	0.0197 (9)	0.0228 (9)	0.0191 (9)	−0.0027 (7)	0.0027 (8)	−0.0110 (8)
C316	0.0260 (12)	0.0252 (11)	0.0207 (11)	−0.0025 (9)	0.0042 (10)	−0.0128 (10)
C317	0.0365 (14)	0.0308 (13)	0.0242 (13)	−0.0109 (11)	0.0053 (11)	−0.0111 (11)
C318	0.0302 (14)	0.0455 (16)	0.0322 (14)	−0.0195 (12)	0.0042 (12)	−0.0146 (13)
C319	0.0234 (13)	0.0471 (16)	0.0297 (14)	−0.0096 (11)	−0.0031 (11)	−0.0149 (13)
C320	0.0313 (13)	0.0270 (12)	0.0276 (13)	−0.0031 (10)	0.0029 (11)	−0.0057 (11)
C401	0.0206 (11)	0.0273 (12)	0.0197 (11)	0.0017 (9)	−0.0044 (9)	−0.0098 (10)
N402	0.0189 (9)	0.0207 (9)	0.0172 (9)	−0.0001 (7)	0.0005 (7)	−0.0073 (8)
N403	0.0196 (9)	0.0249 (10)	0.0209 (10)	0.0022 (7)	0.0003 (8)	−0.0117 (8)
C404	0.0184 (11)	0.0192 (10)	0.0212 (11)	−0.0043 (8)	0.0046 (9)	−0.0090 (9)
S405	0.0198 (3)	0.0288 (3)	0.0196 (3)	−0.0009 (2)	−0.0008 (2)	−0.0123 (2)
S406	0.0205 (3)	0.0367 (3)	0.0281 (3)	0.0005 (2)	0.0018 (2)	−0.0210 (3)
C407	0.0258 (13)	0.0430 (15)	0.0222 (12)	0.0081 (11)	−0.0022 (10)	−0.0115 (12)
C408	0.0196 (11)	0.0305 (12)	0.0223 (12)	0.0038 (9)	−0.0042 (9)	−0.0131 (10)
C409	0.0368 (15)	0.0308 (13)	0.0342 (14)	0.0101 (11)	−0.0020 (12)	−0.0129 (12)
C410	0.0362 (16)	0.0568 (19)	0.0494 (18)	0.0220 (14)	−0.0018 (14)	−0.0283 (16)
C411	0.0249 (14)	0.076 (2)	0.0441 (18)	0.0031 (14)	0.0080 (13)	−0.0248 (17)
C412	0.0310 (15)	0.0505 (17)	0.0374 (16)	−0.0073 (13)	0.0033 (13)	−0.0031 (14)
C413	0.0244 (13)	0.0306 (13)	0.0360 (15)	0.0050 (10)	−0.0042 (11)	−0.0069 (12)
C414	0.0242 (12)	0.0232 (11)	0.0177 (11)	−0.0018 (9)	−0.0004 (9)	−0.0088 (10)
N415	0.0211 (9)	0.0197 (9)	0.0175 (9)	−0.0015 (7)	0.0005 (8)	−0.0085 (8)
C416	0.0227 (11)	0.0195 (11)	0.0201 (11)	−0.0018 (9)	0.0038 (9)	−0.0068 (10)
C417	0.0336 (13)	0.0230 (12)	0.0223 (12)	−0.0011 (10)	0.0073 (10)	−0.0108 (10)
C418	0.0365 (14)	0.0286 (12)	0.0221 (12)	−0.0046 (10)	−0.0004 (11)	−0.0148 (11)
C419	0.0273 (12)	0.0303 (12)	0.0204 (12)	−0.0014 (10)	−0.0039 (10)	−0.0103 (10)
C420	0.0251 (12)	0.0316 (13)	0.0280 (13)	0.0063 (10)	−0.0015 (10)	−0.0136 (11)

### Geometric parameters (Å, º)

**Table:**

Ni1—N202	2.0139 (18)	C218—C219	1.380 (4)
Ni1—N102	2.0173 (17)	C218—H2181	0.945
Ni1—N115	2.1761 (18)	C219—H2191	0.968
Ni1—N215	2.1881 (18)	C220—H2202	0.954
Ni1—S105	2.4062 (6)	C220—H2201	0.963
Ni1—S205	2.4158 (6)	C220—H2203	0.965
Ni2—N302	2.0085 (18)	C301—N302	1.289 (3)
Ni2—N402	2.0156 (18)	C301—C314	1.457 (3)
Ni2—N315	2.1604 (17)	C301—H3011	0.965
Ni2—N415	2.1770 (18)	N302—N303	1.390 (2)
Ni2—S405	2.4202 (6)	N303—C304	1.314 (3)
Ni2—S305	2.4263 (6)	C304—S305	1.712 (2)
C101—N102	1.285 (3)	C304—S306	1.762 (2)
C101—C114	1.461 (3)	S306—C307	1.826 (3)
C101—H1011	0.938	C307—C308	1.504 (3)
N102—N103	1.382 (2)	C307—H3072	0.977
N103—C104	1.312 (3)	C307—H3071	0.989
C104—S105	1.710 (2)	C308—C309	1.396 (3)
C104—S106	1.772 (2)	C308—C313	1.383 (3)
S106—C107	1.819 (2)	C309—C310	1.379 (3)
C107—C108	1.506 (3)	C309—H3091	0.952
C107—H1072	0.994	C310—C311	1.379 (4)
C107—H1071	0.978	C310—H3101	0.960
C108—C109	1.387 (3)	C311—C312	1.382 (4)
C108—C113	1.384 (3)	C311—H3111	0.943
C109—C110	1.383 (4)	C312—C313	1.390 (4)
C109—H1091	0.952	C312—H3121	0.956
C110—C111	1.385 (4)	C313—H3131	0.967
C110—H1101	0.953	C314—N315	1.361 (3)
C111—C112	1.378 (4)	C314—C319	1.386 (3)
C111—H1111	0.962	N315—C316	1.344 (3)
C112—C113	1.389 (3)	C316—C317	1.398 (3)
C112—H1121	0.945	C316—C320	1.489 (3)
C113—H1131	0.968	C317—C318	1.372 (4)
C114—N115	1.367 (3)	C317—H3171	0.955
C114—C119	1.382 (3)	C318—C319	1.384 (4)
N115—C116	1.346 (3)	C318—H3181	0.950
C116—C117	1.398 (3)	C319—H3191	0.960
C116—C120	1.492 (3)	C320—H3202	0.956
C117—C118	1.377 (3)	C320—H3201	0.957
C117—H1171	0.960	C320—H3203	0.959
C118—C119	1.388 (3)	C401—N402	1.287 (3)
C118—H1181	0.976	C401—C414	1.453 (3)
C119—H1191	0.954	C401—H4011	0.969
C120—H1202	0.954	N402—N403	1.391 (2)
C120—H1201	0.968	N403—C404	1.314 (3)
C120—H1203	0.972	C404—S405	1.713 (2)
C201—N202	1.288 (3)	C404—S406	1.754 (2)
C201—C214	1.451 (3)	S406—C407	1.822 (2)
C201—H2011	0.949	C407—C408	1.509 (3)
N202—N203	1.384 (2)	C407—H4072	0.983
N203—C204	1.310 (3)	C407—H4071	0.977
C204—S205	1.712 (2)	C408—C409	1.384 (3)
C204—S206	1.751 (2)	C408—C413	1.385 (3)
S206—C207	1.822 (2)	C409—C410	1.389 (4)
C207—C208	1.499 (3)	C409—H4091	0.964
C207—H2072	0.981	C410—C411	1.374 (4)
C207—H2071	0.979	C410—H4101	0.940
C208—C209	1.385 (3)	C411—C412	1.373 (4)
C208—C213	1.387 (3)	C411—H4111	0.938
C209—C210	1.384 (4)	C412—C413	1.387 (4)
C209—H2091	0.952	C412—H4121	0.942
C210—C211	1.383 (4)	C413—H4131	0.945
C210—H2101	0.945	C414—N415	1.358 (3)
C211—C212	1.377 (4)	C414—C419	1.384 (3)
C211—H2111	0.947	N415—C416	1.347 (3)
C212—C213	1.381 (4)	C416—C417	1.397 (3)
C212—H2121	0.955	C416—C420	1.495 (3)
C213—H2131	0.958	C417—C418	1.374 (3)
C214—N215	1.363 (3)	C417—H4171	0.945
C214—C219	1.389 (3)	C418—C419	1.385 (3)
N215—C216	1.338 (3)	C418—H4181	0.940
C216—C217	1.393 (3)	C419—H4191	0.963
C216—C220	1.499 (3)	C420—H4202	0.966
C217—C218	1.372 (4)	C420—H4201	0.967
C217—H2171	0.955	C420—H4203	0.966
			
N102—Ni1—S105	81.31 (5)	N215—C216—C217	121.4 (2)
N102—Ni1—N115	77.81 (7)	N215—C216—C220	117.4 (2)
S105—Ni1—N115	158.24 (5)	C217—C216—C220	121.2 (2)
N102—Ni1—N202	168.97 (7)	C216—C217—C218	120.2 (2)
S105—Ni1—N202	92.19 (5)	C216—C217—H2171	118.3
N115—Ni1—N202	109.38 (7)	C218—C217—H2171	121.5
N102—Ni1—S205	90.23 (5)	C217—C218—C219	119.3 (2)
S105—Ni1—S205	92.23 (2)	C217—C218—H2181	120.4
N115—Ni1—S205	93.91 (5)	C219—C218—H2181	120.3
N202—Ni1—S205	81.07 (5)	C214—C219—C218	118.1 (2)
N102—Ni1—N215	111.26 (7)	C214—C219—H2191	120.1
S105—Ni1—N215	90.90 (5)	C218—C219—H2191	121.9
N115—Ni1—N215	90.98 (7)	C216—C220—H2202	110.1
N202—Ni1—N215	77.57 (7)	C216—C220—H2201	109.8
S205—Ni1—N215	158.51 (5)	H2202—C220—H2201	109.4
C301—Ni2—N302	23.57 (7)	C216—C220—H2203	111.0
C301—Ni2—S305	104.59 (5)	H2202—C220—H2203	108.6
N302—Ni2—S305	81.02 (5)	H2201—C220—H2203	107.9
C301—Ni2—N315	54.81 (7)	Ni2—C301—N302	38.54 (11)
N302—Ni2—N315	78.22 (7)	Ni2—C301—C314	78.61 (13)
S305—Ni2—N315	158.61 (5)	N302—C301—C314	117.0 (2)
C301—Ni2—C401	161.92 (6)	Ni2—C301—H3011	159.5
N302—Ni2—C401	159.86 (7)	N302—C301—H3011	121.2
S305—Ni2—C401	85.91 (5)	C314—C301—H3011	121.8
N315—Ni2—C401	112.53 (7)	C301—N302—Ni2	117.89 (15)
C301—Ni2—N402	163.65 (7)	C301—N302—N303	116.97 (19)
N302—Ni2—N402	167.55 (7)	Ni2—N302—N303	124.87 (13)
S305—Ni2—N402	89.45 (5)	N302—N303—C304	111.56 (18)
N315—Ni2—N402	111.81 (7)	N303—C304—S305	128.80 (17)
C401—Ni2—N402	23.66 (7)	N303—C304—S306	117.58 (17)
C301—Ni2—S405	88.81 (5)	S305—C304—S306	113.61 (12)
N302—Ni2—S405	92.60 (5)	C304—S305—Ni2	93.50 (7)
S305—Ni2—S405	98.26 (2)	C304—S306—C307	104.61 (11)
N315—Ni2—S405	87.84 (5)	S306—C307—C308	113.51 (16)
C401—Ni2—S405	104.45 (5)	S306—C307—H3072	106.5
C301—Ni2—N415	109.80 (7)	C308—C307—H3072	109.5
N302—Ni2—N415	109.71 (7)	S306—C307—H3071	109.2
S305—Ni2—N415	89.78 (5)	C308—C307—H3071	109.2
N315—Ni2—N415	92.36 (7)	H3072—C307—H3071	108.9
C401—Ni2—N415	54.71 (7)	C307—C308—C309	120.7 (2)
N402—Ni2—S405	80.79 (5)	C307—C308—C313	120.5 (2)
N402—Ni2—N415	78.04 (7)	C309—C308—C313	118.8 (2)
S405—Ni2—N415	157.26 (5)	C308—C309—C310	120.6 (2)
N102—C101—C114	117.05 (19)	C308—C309—H3091	118.7
N102—C101—H1011	122.4	C310—C309—H3091	120.6
C114—C101—H1011	120.5	C309—C310—C311	120.3 (2)
C101—N102—Ni1	118.27 (14)	C309—C310—H3101	119.2
C101—N102—N103	117.29 (17)	C311—C310—H3101	120.6
Ni1—N102—N103	123.88 (13)	C310—C311—C312	119.7 (2)
N102—N103—C104	112.09 (17)	C310—C311—H3111	119.9
N103—C104—S105	128.50 (16)	C312—C311—H3111	120.4
N103—C104—S106	108.80 (15)	C311—C312—C313	120.2 (2)
S105—C104—S106	122.69 (13)	C311—C312—H3121	120.8
C104—S105—Ni1	93.62 (7)	C313—C312—H3121	119.0
C104—S106—C107	104.23 (11)	C312—C313—C308	120.4 (2)
S106—C107—C108	109.55 (17)	C312—C313—H3131	121.0
S106—C107—H1072	108.7	C308—C313—H3131	118.6
C108—C107—H1072	109.7	C301—C314—N315	115.65 (18)
S106—C107—H1071	106.8	C301—C314—C319	121.6 (2)
C108—C107—H1071	112.3	N315—C314—C319	122.8 (2)
H1072—C107—H1071	109.7	C314—N315—Ni2	110.73 (14)
C107—C108—C109	120.2 (2)	C314—N315—C316	118.35 (18)
C107—C108—C113	120.8 (2)	Ni2—N315—C316	130.60 (15)
C109—C108—C113	118.9 (2)	N315—C316—C317	121.1 (2)
C108—C109—C110	120.7 (2)	N315—C316—C320	117.90 (19)
C108—C109—H1091	119.9	C317—C316—C320	121.0 (2)
C110—C109—H1091	119.4	C316—C317—C318	120.2 (2)
C109—C110—C111	119.8 (2)	C316—C317—H3171	119.1
C109—C110—H1101	120.7	C318—C317—H3171	120.7
C111—C110—H1101	119.5	C317—C318—C319	119.2 (2)
C110—C111—C112	120.0 (2)	C317—C318—H3181	119.3
C110—C111—H1111	120.0	C319—C318—H3181	121.6
C112—C111—H1111	120.0	C314—C319—C318	118.4 (2)
C111—C112—C113	119.9 (2)	C314—C319—H3191	120.5
C111—C112—H1121	119.7	C318—C319—H3191	121.1
C113—C112—H1121	120.4	C316—C320—H3202	109.2
C112—C113—C108	120.6 (2)	C316—C320—H3201	110.7
C112—C113—H1131	119.9	H3202—C320—H3201	109.9
C108—C113—H1131	119.5	C316—C320—H3203	109.7
C101—C114—N115	115.53 (18)	H3202—C320—H3203	109.2
C101—C114—C119	121.3 (2)	H3201—C320—H3203	108.3
N115—C114—C119	123.2 (2)	Ni2—C401—N402	38.95 (11)
C114—N115—Ni1	110.65 (13)	Ni2—C401—C414	78.82 (13)
C114—N115—C116	117.90 (18)	N402—C401—C414	117.5 (2)
Ni1—N115—C116	131.37 (15)	Ni2—C401—H4011	160.2
N115—C116—C117	121.3 (2)	N402—C401—H4011	122.0
N115—C116—C120	118.2 (2)	C414—C401—H4011	120.5
C117—C116—C120	120.5 (2)	C401—N402—Ni2	117.39 (15)
C116—C117—C118	120.3 (2)	C401—N402—N403	117.11 (19)
C116—C117—H1171	118.3	Ni2—N402—N403	124.68 (14)
C118—C117—H1171	121.4	N402—N403—C404	111.03 (18)
C117—C118—C119	118.9 (2)	N403—C404—S405	128.97 (17)
C117—C118—H1181	120.2	N403—C404—S406	118.01 (17)
C119—C118—H1181	120.8	S405—C404—S406	113.02 (13)
C118—C119—C114	118.4 (2)	C404—S405—Ni2	93.37 (8)
C118—C119—H1191	121.5	C404—S406—C407	104.55 (11)
C114—C119—H1191	120.1	S406—C407—C408	109.57 (17)
C116—C120—H1202	109.3	S406—C407—H4072	110.5
C116—C120—H1201	111.4	C408—C407—H4072	110.4
H1202—C120—H1201	107.9	S406—C407—H4071	107.9
C116—C120—H1203	109.1	C408—C407—H4071	109.1
H1202—C120—H1203	109.2	H4072—C407—H4071	109.3
H1201—C120—H1203	109.9	C407—C408—C409	120.8 (2)
N202—C201—C214	117.3 (2)	C407—C408—C413	120.5 (2)
N202—C201—H2011	121.2	C409—C408—C413	118.7 (2)
C214—C201—H2011	121.4	C408—C409—C410	120.8 (3)
C201—N202—Ni1	118.51 (15)	C408—C409—H4091	119.6
C201—N202—N203	116.45 (18)	C410—C409—H4091	119.7
Ni1—N202—N203	124.76 (14)	C409—C410—C411	120.0 (3)
N202—N203—C204	111.46 (18)	C409—C410—H4101	119.8
N203—C204—S205	129.06 (17)	C411—C410—H4101	120.3
N203—C204—S206	116.48 (16)	C410—C411—C412	119.8 (3)
S205—C204—S206	114.46 (12)	C410—C411—H4111	119.6
C204—S205—Ni1	93.45 (8)	C412—C411—H4111	120.6
C204—S206—C207	101.78 (11)	C411—C412—C413	120.5 (3)
S206—C207—C208	108.44 (16)	C411—C412—H4121	120.0
S206—C207—H2072	107.2	C413—C412—H4121	119.5
C208—C207—H2072	109.9	C412—C413—C408	120.3 (2)
S206—C207—H2071	107.8	C412—C413—H4131	119.1
C208—C207—H2071	111.0	C408—C413—H4131	120.7
H2072—C207—H2071	112.4	C401—C414—N415	115.91 (19)
C207—C208—C209	120.9 (2)	C401—C414—C419	120.9 (2)
C207—C208—C213	120.2 (2)	N415—C414—C419	123.2 (2)
C209—C208—C213	118.9 (2)	C414—N415—Ni2	110.05 (14)
C208—C209—C210	120.5 (3)	C414—N415—C416	117.96 (19)
C208—C209—H2091	119.5	Ni2—N415—C416	131.48 (15)
C210—C209—H2091	120.0	N415—C416—C417	121.0 (2)
C209—C210—C211	119.9 (3)	N415—C416—C420	117.8 (2)
C209—C210—H2101	119.1	C417—C416—C420	121.2 (2)
C211—C210—H2101	121.0	C416—C417—C418	120.8 (2)
C210—C211—C212	120.0 (2)	C416—C417—H4171	118.8
C210—C211—H2111	119.6	C418—C417—H4171	120.4
C212—C211—H2111	120.4	C417—C418—C419	118.4 (2)
C211—C212—C213	120.0 (3)	C417—C418—H4181	120.4
C211—C212—H2121	120.4	C419—C418—H4181	121.2
C213—C212—H2121	119.6	C418—C419—C414	118.7 (2)
C208—C213—C212	120.7 (2)	C418—C419—H4191	121.6
C208—C213—H2131	119.3	C414—C419—H4191	119.8
C212—C213—H2131	120.0	C416—C420—H4202	109.4
C201—C214—N215	115.70 (19)	C416—C420—H4201	108.8
C201—C214—C219	121.3 (2)	H4202—C420—H4201	109.5
N215—C214—C219	123.0 (2)	C416—C420—H4203	110.6
Ni1—N215—C214	110.67 (14)	H4202—C420—H4203	108.7
Ni1—N215—C216	131.22 (15)	H4201—C420—H4203	109.9
C214—N215—C216	118.07 (19)		
